# Assessment of a new genomic classification system in acute myeloid leukemia with a normal karyotype

**DOI:** 10.18632/oncotarget.23575

**Published:** 2017-12-22

**Authors:** Jae-Sook Ahn, Hyeoung-Joon Kim, Yeo-Kyeoung Kim, Seung-Shin Lee, Seo-Yeon Ahn, Sung-Hoon Jung, Deok-Hwan Yang, Je-Jung Lee, Hee Jeong Park, Ja-Yeon Lee, Seung Hyun Choi, Chul Won Jung, Jun-Ho Jang, Hee Je Kim, Joon Ho Moon, Sang Kyun Sohn, Yoo Jin Lee, Jong-Ho Won, Sung-Hyun Kim, Zhaolei Zhang, TaeHyung Kim, Dennis Dong Hwan Kim

**Affiliations:** ^1^ Hematology-Oncology, Chonnam National University Hwasun Hospital, Jeollanam-do, Korea; ^2^ Genomic Research Center for Hematopoietic Diseases, Chonnam National University Hwasun Hospital, Jeollanam-do, Korea; ^3^ Division of Hematology-Oncology, Samsung Medical Center, Seoul, Korea; ^4^ Department of Hematology, The Catholic University of Korea, Seoul, Korea; ^5^ Department of Hematology-Oncology, Kyungpook National University Hospital, Seoul, Korea; ^6^ Department of Hematology-Oncology, Soon Chun Hyang University Hospital, Seoul, Korea; ^7^ Department of Hematology-Oncology, Dong-A University College of Medicine, Busan, Korea; ^8^ Department of Computer Science, University of Toronto, Toronto, ON, Canada; ^9^ The Donnelly Centre for Cellular and Biomolecular Research, University of Toronto, Toronto, ON, Canada; ^10^ Department of Molecular Genetics, University of Toronto, Toronto, ON, Canada; ^11^ Department of Medical Oncology and Hematology, Princess Margaret Cancer Centre, University of Toronto, Toronto, ON, Canada

**Keywords:** genomic classification, AML, next generation sequencing, normal karyotype, prognosis

## Abstract

This study was performed to assess if a recently recommended genomic classification is predictive in patients with normal-karyotype (NK) acute myeloid leukemia (AML). A total of 393 patients were included. Analysis of genetic mutations was performed using targeted resequencing with an Illumina Hiseq 2000. We identified driver mutations across 40 genes, with one or more driver mutations identified in 95.7% of patients. The molecular subclassification was as follows: 34.6% patients (n = 136) with AML with the *NPM1* mutation, 10.7% (n = 42) with AML with mutated chromatin or RNA-splicing genes or both, 1.5% (n = 6) with AML with *TP53* mutations, 13.5% (n = 53) with AML with biallelic *CEBPA* mutations, 2.0% (n = 8) with AML with *IDH2-R1*72 mutations and no other class-defining lesion, 29.5% (n = 116) with AML with driver mutations but no detected class-defining lesion, 4.3% (n = 17) with AML with no detected driver mutation, and 3.8% (n = 15) patients with AML who met the criteria for ≥2 genomic subgroups. The 5-year overall survival and relapse rate of subgroup in AML with mutated chromatin, RNA-splicing genes, or both was 11.6% (95% CI = 1.4–21.8%) and 71.4% (95% CI = 45.7–86.5%), respectively. This study suggests that the recently recommended genomic classification is an appropriate and replicable categorization system in the NK AML population. The subgroup of AML with mutated chromatin, RNA-splicing genes, or both showed extremely poor survival in NK-AML; thus, a novel approach is needed to improve their prognosis.

## INTRODUCTION

Acute myeloid leukemia (AML) is a genetically heterogeneous disease. In the past two decades, clonal chromosomal aberrations have been recognized as the most important marker for prognostication in AML patients [1]. The 2008 WHO classification suggested several subtypes of AML with recurrent genetic abnormalities, among which individuals with mutated *NPM1* and *CEBPA* were proposed as provisional entities [2]. Many studies have described the significance of cytogenetic and/or molecular abnormalities in patients with AML since European LeukemiaNet recommended a standardized reporting system of AML classification based on cytogenetic and molecular genetic abnormalities in 2010 [3]. A revised WHO classification system in 2016 incorporated emerging data into the system and classified the group with mutations in *NPM1* and biallelic mutations of *CEBPA* as a separate AML subtype [4]. In addition, the provisional category of AML with mutated *RUNX1* was added to the *de novo* AML classification.

Several studies have attempted to adopt the molecular genetic classification to correlate clinical outcome in AML patients in a group with a specific cytogenetic subgroup [4–7]. More recently, Papaemmanuil *et al*. [8] reported that genomic classification in AML can improve the classification of AML subtype according to prognosis, and can distinguish each subtype of AML based on their driver mutation and underlying pathway to induce leukemogenesis. However, this classification has yet to be validated in an independent group of patients with AML. A previous study could have excluded some elderly patients with AML given that it was conducted in the context of a prospective clinical trial that usually excludes a large proportion of patients from enrollment due to comorbidities or age issues. However, in the real world, the proportion of elderly AML patients is increasing, which might have been underrepresented in the previous study by Papaemmanuil *et al* [8].

Thus, in this study, we evaluated whether the recommended genomic classification of AML is relevant to patients with AML, particularly in the subgroup with a normal karyotype (NK), including elderly AML patients.

## RESULTS

### Frequency of mutations and genomic classification in NK-AML

**Figure 1:**
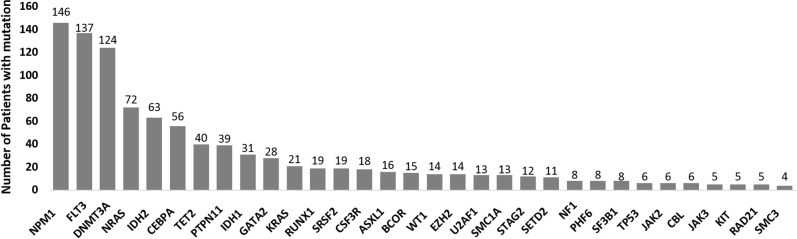
Frequently detected mutations in 393 patients with normal-karyotype acute myeloid leukemia (NK-AML) at diagnosis

**Figure 2:**
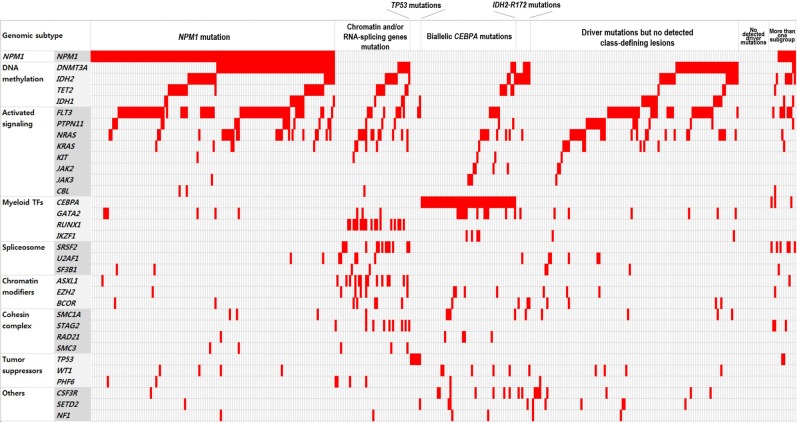
Schematic representation of the mutational status of patients with NK-AML at diagnosis Colored grids indicate mutation-positive subjects.

In total, 1,060 driver mutations were identified, involving 40 genes in 393 patients. Frequently detected mutations in 393 patients at diagnosis were described in Figure 1. At least one driver mutation was observed in 376 of 393 (95.7%) patients, and two or more driver mutations in 309 (78.6%) (Supplementary Figure 1). The mutations and their associations with other mutations are presented in Figure 2. The prevalence rates for the frequently observed mutations were as follows: *NPM1*^mut^ (*n* = 146, 37.2%), *FLT3*^mut^ (*n* = 137, 34.9%), *DNMT3A*^mut^ (*n* = 124, 31.6%), *IDH1/2*^mut^ (*n* = 94, 23.9%), *NRAS*^mut^ (*n* = 72, 18.3%), *CEBPA* double mutations (*n* = 56, 14.2%), and *TET2*^mut^ (*n* = 40, 10.2%). We used a lollipop plot to display frequently detected mutations (Supplementary Figure 2). Based on the genomic classification criteria [8], the patients were classified as follows: 136 (34.6%) with *NPM1* mutations, 42 (10.7%) with mutated chromatin modifiers and/or RNA-splicing genes, 6 (1.5%) with *TP53* mutations, 53 (13.5%) with biallelic *CEBPA* mutations, 8 (2.0%) with *IDH2*-R172 mutations and no other class-defining lesions, 116 (29.5%) with driver mutations but no detected class-defining lesions, 17 (4.3%) with no detected driver mutations, and 15 (3.8%) patients who met the criteria of more than one genomic subgroup categorized above (Table 1). AML with myeloid dysplasia-related changes was observed in 28 (7.1%), and secondary AML was detected in 29 (7.4%) of 393 patients. The frequencies of AML with myeloid dysplasia-related changes and secondary AML were not different between the classifications (all, p > 0.05).

**Table 1:** Patient characteristics and genomic subgroups

	No. of patients (%)	*NPM1* mutation	Chromatin and/or RNA-splicing genes mutation	*TP53* mutations	Biallelic *CEBPA* mutations	*IDH2*-*R172* mutations and no other class-defining lesion	Driver mutations but no detected class-defining lesion	No detected driver mutation	More than one subgroup
No. of patients (%)	393	136 (34.6)	42 (10.7)	6 (1.5)	53 (13.5)	8 (2.0)	116 (29.5)	17 (4.3)	15 (3.8)
Median age, years (range)	53 (15–83)	54 (15–84)^*^	59(19–76) ^**^	39 (22–54)	40 (15–72)^**^	58 (51–64)^*^	50 (17–83)	41 (23–69)	59 (16–75)
Sex, male (%)	201/393 (51.2)	58/136 (42.6) ^*^	32/42 (76.2)^*^	3/6 (50.0)	22/53 (41.5)	6/8 (75.0)	62/116 (53.4)	8/17 (47.1)	10/15 (66.7)
WBC, median, × 10^9^/L (range)	25.6 (0.3–397.2)	41.7 (0.9–384.0)^*^	12.6 (0.5–397.2)	8.8 (2.0–120.7)	28.5 (4.5–333.2)	1.6 (0.5–8.6)^*^	23.7 (0.5–279.0)	2.5 (0.3–121.0) ^*^	13.9 (1.2–142.0)
Bone marrow blast, % (range)	70 (2–100)	79 (3–100)^*^	60 (10–100)^*^	48 (16–100)	70 (16–100)	72 (56–100)	68 (2–100)	79 (12–100)	69 (23–100)
CR achievement, (%)	325 (82.7)	118/136 (86.8)	26/42 (61.9)^**^	5/6 (83.3)	50/53 (94.3)^*^	7/8 (87.5)	91/116 (78.4)	16/17 (94.1)	12/15 (80.0)
Allogeneic SCT at 1^st^ CR, (%)	129/393 (32.8)	46/136 (33.8)	6/42 (14.2) ^*^	3/6 (50.0)	30/53 (56.6)^*^	3/8 (37.5)	33/116 (28.4)	6/17 (35.3)	2/15 (13.3)
5-year relapse rate (95% CI)	42.5 (36.8–48.2)	40.3 (30.4–50.0)	71.4 (45.7–86.5)^*^	20.0 (0.4–61.2)	21.3 (10.8–34.1)^*^	21.4 (0.3–67.3)	53.9 (41.8–64.5) ^*^	43.8 (18.7–66.5)	33.3 (9.2–60.3)
5-year EFS (95% CI)	32.8 (27.9–37.7)	41.6 (32.6–50.6)^*^	5.3 (0–12.3) ^**^	66.7 (29.1–104.3)	52.6 (38.5–66.7)^*^	37.5 (0–77.3)	20.4 (12.4–28.4) ^**^	29.4 (7.6–51.4)	40.0 (15.3–64.7)
5-year OS (95% CI)	37.8 (32.5–43.1)	49.3 (40.1–58.5)^*^	11.6 (1.4–21.8) ^**^	50.0 (10.0–90.0)	58.4 (44.1–72.7)^*^	56.3 (17.3–95.3)	24.3 (15.6–32.9) ^**^	29.4 (7.6–51.1)	40.0 (15.3–64.7)

Abbreviations: CR, complete remission; SCT, stem cell transplantation; CI, confidence interval; EFS, event-free survival; OS, overall survival; WBC, white blood cells.

^*^ The range of *P* values is 0.001 ≤ *P* < 0.05 versus all other patients.

^**^ The range of *P* values is < 0.001 versus all other patients.

### Clinical features according to genomic subtype based on genomic classification of AML

Patient characteristics according to genomic classifications are described in Table 1. The patients with biallelic *CEBPA* mutations were observed to be younger (*p* < 0.001). However, the patients with *NPM1* mutations (*p* = 0.010), chromatin and/or RNA splicing gene mutations (*p* < 0.001), and *IDH2*-R172 mutations and no other class-defining lesions (*p* = 0.004) were older compared with the rest of the cohort. *NPM1* mutations were observed more frequently in females (78/136, 57.4%, *p* = 0.014) and chromatin and/or DNA splicing gene mutation were frequent in males (32/42, 76.2%, *p* = 0.001). *NPM1* mutations were associated with high WBC counts (*p* = 0.010) and increased bone marrow blasts (*p* = 0.033) compared with the rest of the cohort. Patients with *IDH2*-R172 mutations and no other class-defining lesions were associated with low WBC counts, and patients with chromatin and/or DNA splicing genes mutation were associated with decreased bone marrow blasts (*p* = 0.023).

### Complete remission rate according to AML subtype based on the genomic classification

Patients had received induction chemotherapy using a standard protocol [3-day course of anthracycline with a simultaneous 7-day course of cytosine arabinoside (Ara-C) or N^4^-behenoyl-1-b-d-arabinofuranosylcytosine (BHAC)]. Idarubicin was administered daily at a dose of 12 mg/m^2^ or daunorubicin was administered at a dose of 60 mg/m^2^ on three consecutive days. Ara-C was administered daily at a dose of 100 mg/m^2^ and BHAC at a dose of 300 mg/m^2^ on seven consecutive days. In all, 231 patients were treated with idarubicin + Ara-C, 71 patients were treated with idarubicin + BHAC, and 91 patients received daunorubicin + Ara-C induction chemotherapy. Of 393 patients, 273 (69.5%) achieved complete remission (CR) after first induction chemotherapy. Eighty-two patients received second induction chemotherapy (50 patients received first induction regimen, 12 patients received mitoxantrone based induction and, 20 patients received fludarabine based induction) and 44 patients achieved CR after second induction chemotherapy. Eight of 22 patients achieved CR after third induction chemotherapy.

Of the 393 patients, 325 (82.7%) achieved CR. CR rates varied depending on the genomic subgroups (61.9–97.2%). The CR rate for each subgroup was as follows: 86.8% (118/136, 95% confidence interval [CI] = 81.0–92.5) of patients with *NPM1* mutations, 61.9% (26/42, 95% CI = 46.6–77.2) of patients with mutated chromatin and/or RNA-splicing genes, 83.3% (5/6, 95% CI = 40.5–100) of patients with *TP53* mutations, 94.3% (50/53, 95% CI = 40.5–100) of patients with biallelic *CEBPA* mutations, 87.5% (7/8, 95% CI = 57.9–100) of patients with *IDH2*-R172 mutations and no other class-defining lesions, 78.4% (91/116, 95% CI = 70.6–85.9) of patients with driver mutations but no detected class-defining lesions, 94.1% (16/17, 95% CI = 82.7–100) of patients with no detected driver mutation, and 80.0% (12/15, 95% CI = 57.1–100) of patients meeting criteria of more than one subgroup. The CR rates of the subgroup with biallelic *CEBPA* mutations were higher than the remaining cohorts (94.3% vs. 80.9%, odds ratio = 3.939, 95% CI = 1.191–13.02; p = 0.016). The CR rates of the subgroup with mutated chromatin and/or RNA-splicing genes were significantly lower than in the rest of the cohort (61.9% vs. 85.2%, odd ratios = 0.283, 95% CI = 0.142–0.563; *p* < 0.001).

### Survival rate according to AML subtype based on genomic classification

**Figure 3:**
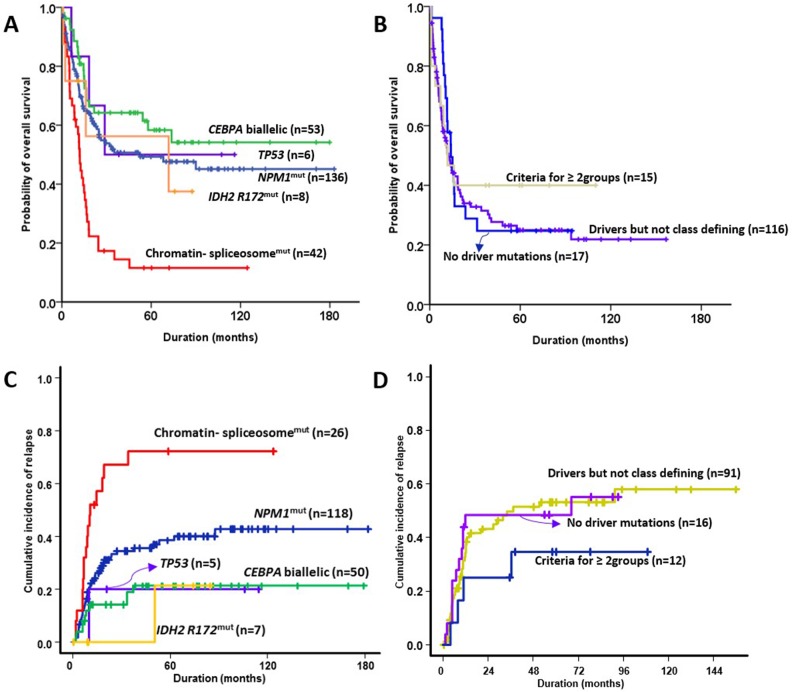
Prognostic impact in NK-AML according to genomic classifications **(A, B)** overall survival, and **(C, D)** relapse incidence.

Allogeneic stem cell transplantation (SCT) was performed in 32.8% (129/393) of the patients at first CR. Allogeneic SCT was undertaken in the first CR in 56.6% of patients with biallelic *CEBPA* mutations, (OR = 1.470, 95% CI = 1.207–1.790; *p* = 0.001) and in 14.2% (6/42) of the subgroup with mutated chromatin and/or RNA-splicing genes (OR = 0.309, 95% CI = 0.127–0.754; *p* = 0.007) compared to the rest of the cohort. The 5-year overall survival (OS) and 5-year relapse rates for each subgroup were as follows: 49.3% (95% CI = 40.1–58.5) and 40.3% (95% CI = 30.4–50.0) in patients with *NPM1* mutations, 11.6% (95% CI = 1.4–21.8) and 71.4% (95% CI = 45.7–86.5) in patients with mutated chromatin and/or RNA-splicing genes, 50.0% (95% CI = 10.0–90.0) and 20.0% (95% CI = 0.4–61.2) of patients with *TP53* mutations, 58.4% (95% CI = 44.1–72.7) and 21.3% (95% CI = 10.8–34.1) of patients with biallelic *CEBPA* mutations, 56.3% (95% CI = 17.3–95.3) and 21.4% (95% CI = 0.3–67.3) of patients with *IDH2*-R172 mutations and no other class-defining lesion, 24.3% (95% CI = 15.6–32.9) and 53.9% (95% CI = 41.8–64.5) of patients with driver mutations but no detected class-defining lesion, 29.4% (95% CI = 7.6–51.1) and 43.8% (95% CI = 18.7–66.5) of patients with no detected driver mutations, and 40.0% (95% CI = 15.3–64.7) and 33.3% (95% CI = 9.2–60.3) of patients that met the criteria of more than one subgroup (Figure 3). The group with biallelic *CEBPA* mutations showed a statistically favorable overall survival (OS) (p = 0.004) and lower rate of relapse (p = 0.001) than the others. However, the groups with mutated chromatin and/or RNA-splicing genes (p< 0.001 and 0.001, respectively) and driver mutations but no detected class-defining lesion (p< 0.001 and 0.010, respectively) showed inferior OS and higher relapse rates compared with the remaining cohorts. The group with *NPM1* mutations showed a favorable OS (*p* = 0.001) compared with the others.

Of the 393 patients, 129 patients underwent allogeneic SCT. The 5-year OS was 56.7% (95% CI = 47.7–65.7) in patients who underwent allogeneic SCT. There were no significant differences in the survival rate according to the genomic subgroups among the patients who underwent transplantation compared to the remaining cohorts. However, the subgroup with mutated chromatin and/or RNA-splicing genes showed a trend for inferior survival (*p* = 0.161; Supplementary Figure 3).

Of the 393 patients, we sub-analyzed survival by censoring the allogeneic SCT (Supplementary Figure 4). The 5-year OS was 31.3% (95% CI = 24.8–37.8). The group with biallelic *CEBPA* mutations showed a statistically favorable OS (p = 0.009) and a lower rate of relapse (p = 0.048) than the others. However, the groups with mutated chromatin and/or RNA-splicing genes (p = 0.003 and 0.003, respectively) showed inferior OS and higher relapse rates compared with the remaining cohorts. Driver mutations but no detected class-defining lesion (*p* < 0.001) showed inferior OS and the group with *NPM1* mutations showed a favorable OS (*p* < 0.001) compared with the others. These results were similar to those of patients undergoing allogeneic SCT who were not censored at the time of transplantation.

In summary, the subgroup with biallelic *CEBPA* mutations showed the highest CR rates and best OS. The subgroup with mutated chromatin and/or RNA-splicing genes was statistically significantly worst in OS and CR achievement.

## DISCUSSION

In this study, we evaluated the clinical relevance of the genomic classification system using targeted deep sequencing and examined its prognostic implication in 393 patients with NK-AML. The genomic classification system is useful for stratifying NK-AML patients according to their prognosis. The results in this study, confined to a subgroup of AML patients with a normal karyotype, showed similar survival patterns to those reported in the study by Papaemmanuil [8]. The subgroup in AML with mutated chromatin, RNA-splicing genes, or both showed extremely poor survival, whereas the group with *TP53* mutations showed somewhat better outcomes than in a previous paper, although the number of patients with *TP53* mutations was much smaller (1.5%) than that reported in a NK-AML population [8].

Papaemmanuil *et al*. presented a new genetic approach to AML classification with prognostic implications [8]. The classification included the known cytogenetic lesions together with *NPM1*, *FLT3*-ITD, and *CEBPA*. In addition, the classification incorporated *TP53*, chromatin-spliceosome mutations, and *IDH2 R172* mutations because they are common and have strong influences on clinical outcomes. The panels of targeted gene sequencing in our study covered nearly all of the frequently detected driver mutations in a previous study [8]. However, our study focused exclusively on the subgroup with NK-AML. Thus, there were some differences in the distributions of subgroups. *NPM1* mutation (35% vs. 27%), biallelic *CEBPA* mutations (14% vs. 4%), and driver mutations with no detected class-defining lesion (30% vs. 11%) were observed more commonly in our cohort than in prior genomic classifications, because such mutations were observed more frequently in NK AML and the population with NK AML fundamentally excluded cytogenetic abnormalities.

*TP53* mutations in NK-AML were only observed in 1.5% of all NK-AML patients. In the previous genomic classification, *TP53* mutations were included in the genomic subgroup of AML with *TP53* mutations, chromosomal aneuploidy, or both. In fact, *TP53* mutations not accompanying a complex karyotype were observed in only 17 (1.1%) patients of 1,540 in the original genomic classification result [8]. In our cohort, the OS and relapse risk in *TP53* mutations in NK AML were 50% and 20%, respectively. However, only six patients were included in the *TP53* mutated group and three of the six patients underwent allogeneic SCT. Clearly, that is a very small number of patients to reach any clear conclusion on this issue of the prognostic relevance of the *TP53* mutation group in NK-AML (Supplementary Figure 2).

The subgroup in AML with mutated chromatin and RNA-splicing genes were older, with lower bone marrow blasts. This subgroup showed a low CR rate and poor overall survival. Similar results were observed in the original genomic classification [8]. Chromatin and/or RNA splicing genes mutations are frequently observed in myelodysplastic syndrome and secondary AML [8–10]. This subgroup is classified in the intermediate I or intermediate II risk group according to the European LeukemiaNet recommendations [3, 8]. However, the 5-year OS in that subgroup was only 11.6% (HR = 0.490, 95% CI = 0.343–0.701) and the relapse rate was 71.4% (HR = 2.378, 95% CI = 1.442–3.921). The treatment outcome in the AML subgroup with mutated chromatin or RNA-splicing genes was extremely poor and showed a similar outcome to that in the adverse cytogenetic risk group [1]. That subgroup included older patients and a low CR rate was observed; such factors, consequently, may influence the treatment chance of allogeneic SCT. Our results showed that NK-AML with mutated chromatin and RNA-splicing genes should be classified as a distinct adverse risk group. This subgroup requires innovative treatment interventions to improve outcomes, such as the early incorporation of targeted therapy during AML treatment and post-transplant targeted maintenance/intervention.

In conclusion, the new genomic approach to AML classification with prognostic implications is reproducible in the population of NK-AML patients. In clinical aspects, the number of cases with *TP53* mutations in NK-AML was very small and may not be an inferior prognostic factor. To clearly demonstrate the role of *TP53* mutations in NK-AML, a clinical study involving a large number of patients is important. The subgroup in AML with mutated chromatin and RNA-splicing genes, or both, showed extremely poor prognoses in terms of clinical features and treatment results. That subgroup needs novel approaches to improve their results.

## MATERIALS AND METHODS

### Patients and methods

In total, 393 patients diagnosed with NK-AML from October 1998 to October 2014 at seven participating institutes were included in the study. All of the patients met the following eligibility criteria: age ≥ 15 years, a diagnosis of NK-AML confirmed by conventional cytogenetic analysis, and treatment with induction chemotherapy using a standard protocol (a 3-day course of anthracyclines with a 7-day course of cytosine arabinoside). Patients who achieved CR received consolidation chemotherapy with or without allogeneic SCT, depending upon the availability of a matched related or unrelated donor. Genetic factors were not considered when choosing allogeneic SCT as a consolidation treatment. We provide a flowchart on patients’ selections in Supplementary Figure 5. Written informed consent was obtained from all of the subjects for the genetic analysis of samples taken at the time of the initial diagnosis. This study was approved by the Institutional Review Board of the Chonnam National University Hwasun Hospital, Korea (IRB number: CNUHH-2014-153).

HLA typing at low resolution was used for HLA-A, -B, -C, and -DR donor searches before 2006, whereas it was at high resolution after 2006. Sibling donors with HLA-A, -B, -C, and -DRB1 8/8 matched related donors were defined as matched related donors. For unrelated transplantations, HLA-A, -B, -C, and DRB1 8/8 matched unrelated donors were defined as matched unrelated donors and transplantation from HLA- A, -B, -C, and –DRB1 7/8 or 6/8 matched unrelated donors was defined as mismatched unrelated donors. The conditioning regimens were classified as myeloablative if total body irradiation ≥ 8 Gy (*n* = 42), oral busulfan ≥ 9 mg/kg, or intravenous busulfan ≥ 7.2 mg/kg (*n* = 67) was included in the conditioning regimen, whereas other conditioning regimens were classified as reduced-intensity conditioning regimens (*n =* 20). In most cases, GVHD prophylaxis was a cyclosporine-based regimen (CSA; *n* = 85), and 44 patients received an FK506-based regimen. We describe the characteristics of the 129 patients who received allogeneic SCT in Supplementary Table 2.

### Genetic analyses and grouping

Cryopreserved bone marrow or peripheral blood samples taken at diagnosis were archived. Genomic DNA was extracted using QIAamp DNA blood mini-kits (Qiagen, Valencia, CA, USA) according to the manufacturer's protocol. Genetic profiling included the targeted deep sequencing of 92 genes, which had been selected based on recurrent driver mutations from previous studies and our own exome sequencing (currently unpublished) [7, 8]. Agilent custom probes were designed to cover the entire exon regions of targeted genes (92 genes) and sequenced with the manufacturer's protocol using an Illumina HiSeq 2000 sequencer (Supplementary Table 1). First, all sequenced reads were mapped to hg19 using Burrows-Wheeler [11]. Then, the mapped PCR duplicates were marked using the Picard toolkit [12]. We then realigned indels, fixed mate information, and recalibrated the base scroe using the Genome Analysis Toolkit [13]. A different approach was taken with the somatic variants, depending on the availability of control samples. When the control sample was available, we first used Fisher's exact test on a 2 × 2 contingency table consisting of reference and alternative alleles from tumor and control samples to filter out possible germline variants and noise from the experimental procedure. A minimum threshold of 3% was used (p < 0.001). Variants resulting from the above procedures were further assessed using dbSNP135, esp6500, ClinVar, and COSMIC databases [14–16]. When a control sample was not available, we used the Shearwater algorithm to refer candidates for somatic variants as well as somatic variants identified from paired samples.

The genomic classification in AML was followed, as described previously [8]. Particularly, the classification in AML with mutated chromatin, RNA-spicing genes, or both was defined with one or more driver mutations in *RUNX1*, *ASXL1*, *BCOR*, *STAG2*, *EZH2*, *SRSF2*, *SF3B1*, *U2AF1*, or *ZRSR2*.

### Endpoints of response and survival

CR was defined as the presence of a morphologically normal marrow with fewer than 5% blasts, no evidence of extramedullary leukemia, and recovery of the peripheral platelet count to ≥ 100×10^9^/L and neutrophil count to ≥ 1.0×10^9^/L, for at least 4 weeks, in the absence of chemotherapy. The relapse rate was defined as the time from attainment of remission to the date of relapse in all of the patients who achieved CR, considering competing events of death without relapse. Non-relapse mortality was defined as death occurring in the absence of relapse. Event-free survival (EFS) was defined as the time from commencement of induction chemotherapy to the date of death from any cause, relapse, or non-achievement of CR, whichever occurred first. OS was defined as the time from beginning induction chemotherapy to the date of the last follow-up, or death from any cause. Patients undergoing allogeneic SCT were not censored at the time of transplantation.

### Statistical analysis

Descriptive statistics are presented as frequencies with percentages for categorical variables, and as medians with ranges for continuous variables. The χ^2^ test was used to compare differences in distributions of categorical data and Student's *t*-test was used to evaluate the significance of differences in continuous variables. EFS and OS were estimated using Kaplan–Meier survival curves; differences among groups were compared using the log-rank test. Because allogeneic SCT is a time-dependent event, time-dependent Cox regression was performed with allogeneic SCT as a time-dependent factor for survival analysis. The prognostic impact of various risk factors on EFS and OS was evaluated in univariate analyses using a time-dependent Cox proportional hazard model. Relapse rates were calculated using a cumulative incidence method considering competing risks, and Gray's test was used for comparisons [9]. *P*-values < 0.05 were considered statistically significant. Hazard ratios (HRs) and 95% CIs were estimated using a predetermined reference risk value of unity. All of the statistical analyses were performed using the SPSS software (ver. 21.0; SPSS Inc., Chicago, IL, USA) and EZR software, using the ‘R’ language (available at http://www.jichi.ac.jp/saitama-sct/SaitamaHP.files/statmedEN.html) [17].

## SUPPLEMENTARY MATERIALS FIGURES AND TABLES




