# Supplementary material for: Assessment of a new genomic classification system in acute myeloid leukemia with a normal karyotype
Source: Oncotarget. 2017 Dec 22;9(4):4961–8. doi: 10.18632/oncotarget.23575 (PMC5797026; doi:10.18632/oncotarget.23575)
Supplement: Supplementary file 1 [file oncotarget-09-4961-s001.pdf]

Assessment of a new genomic classification system in acute myeloid leukemia with a normal karyotype

SUPPLEMENTARY MATERIALS

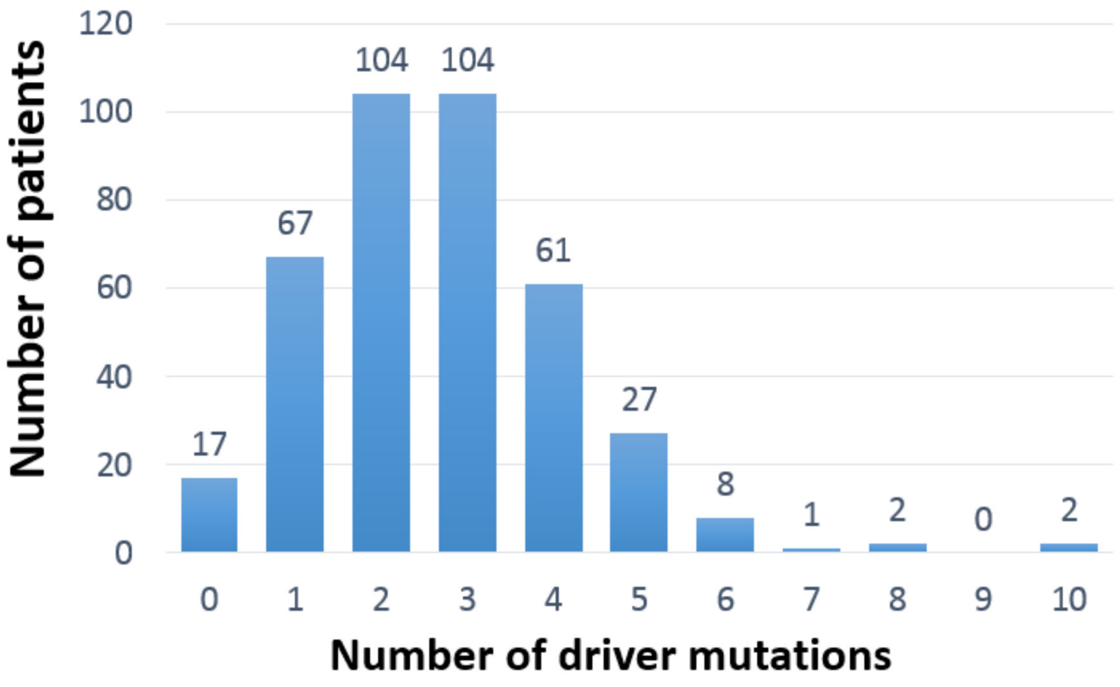

Supplementary Figure 1: Bar plot indicating number of driver events by patient for all patients.

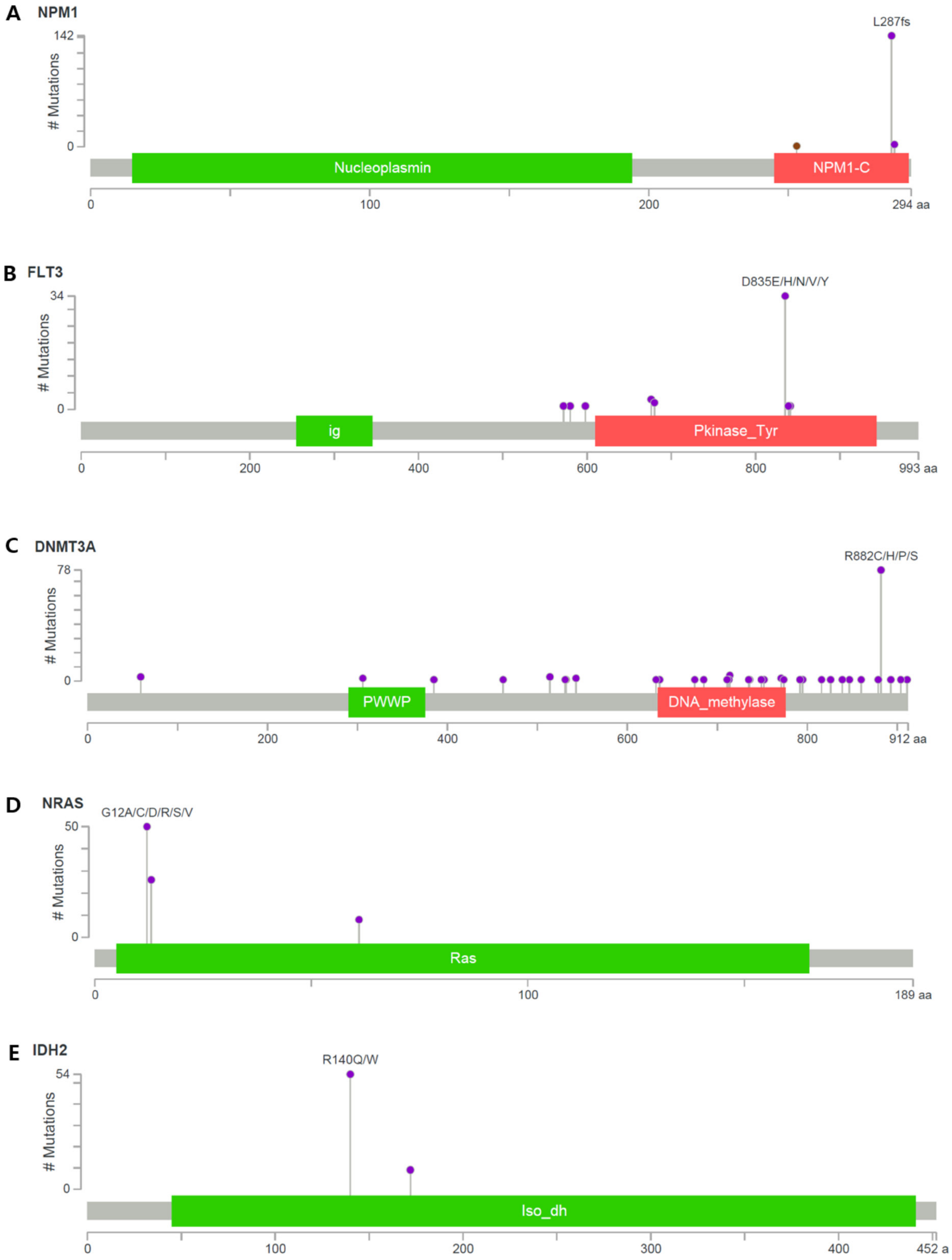

Supplementary Figure 2 ***(Continued)*****:** Lollipop plot of the most frequently detected mutations **(A-V)** including TP53 **(W)**. We described the location of *TP53* mutation in **W(a)**; out cohort, **W(b)**; Metzeler's [Blood 2016] and, **W(c)**; Papaemanuil's data [NEJM 2016].

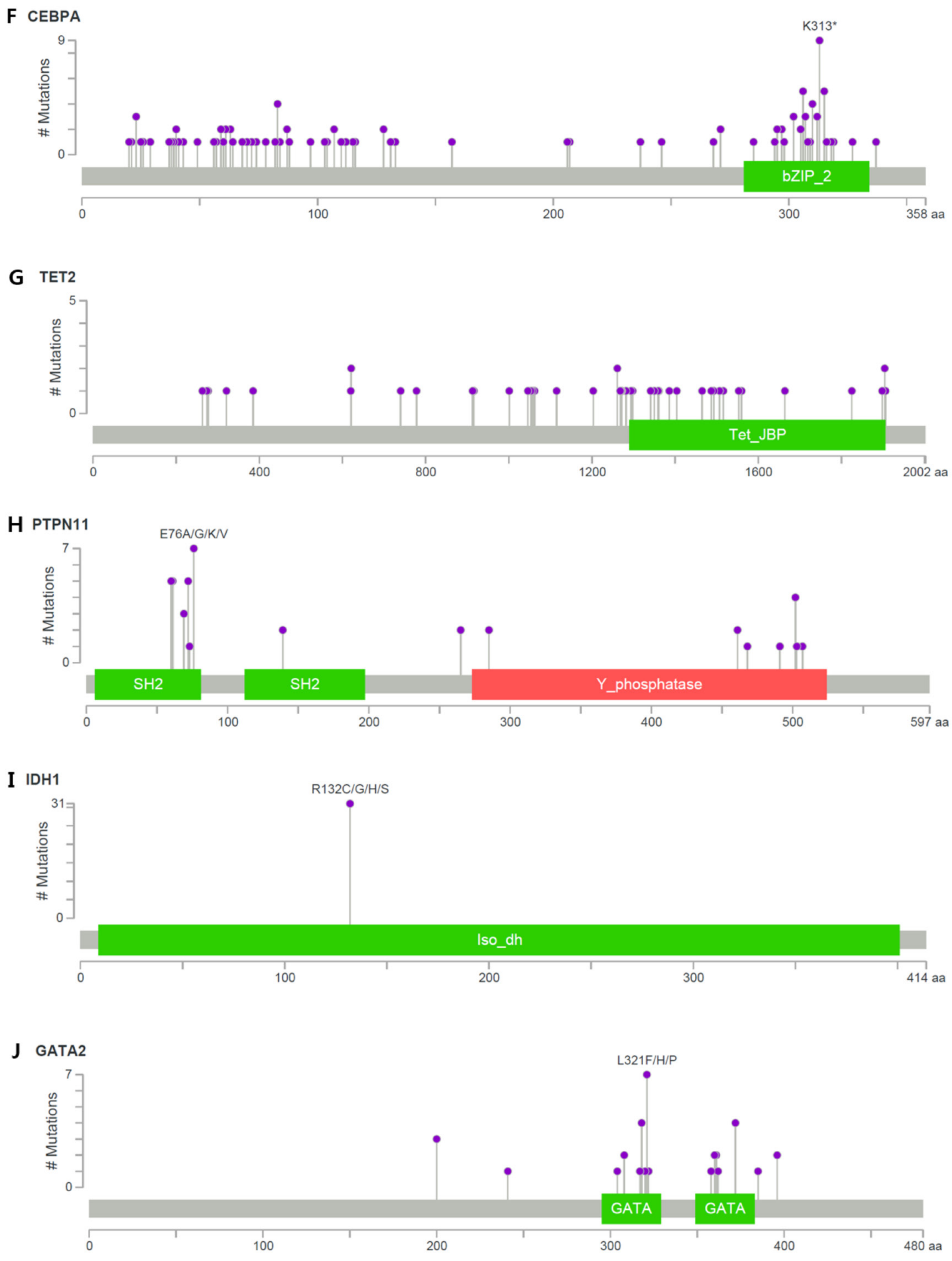

Supplementary Figure 2: Lollipop plot of the most frequently detected mutations **(A-V)** including TP53 **(W)**. We described the location of *TP53* mutation in **W(a)**; out cohort, **W(b)**; Metzeler's [Blood 2016] and, **W(c)**; Papaemanuil's data [NEJM 2016]. ***(Continued)***

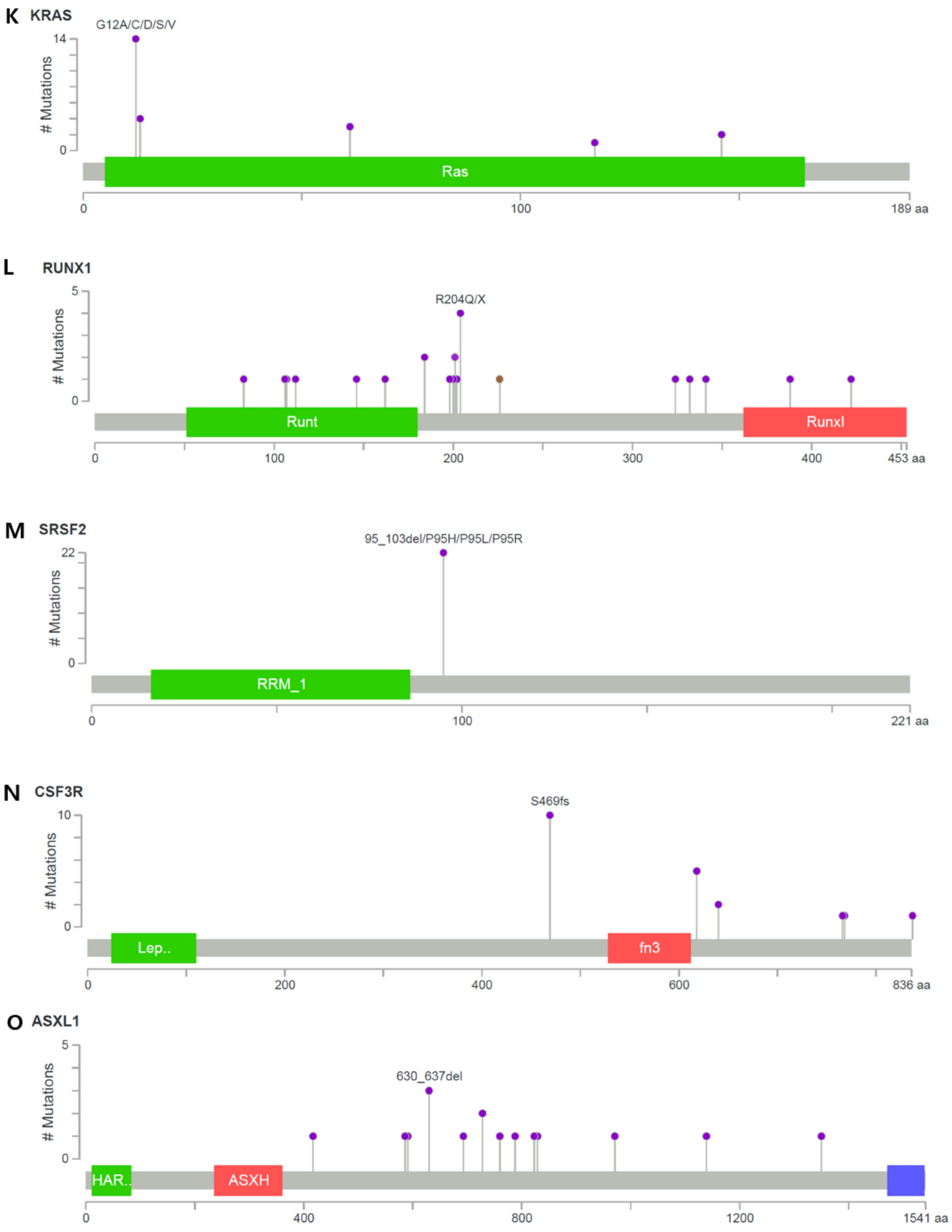

Supplementary Figure 2: Lollipop plot of the most frequently detected mutations **(A-V)** including TP53 **(W)**. We described the location of *TP53* mutation in **W(a)**; out cohort, **W(b)**; Metzeler's [Blood 2016] and, **W(c)**; Papaemanuil's data [NEJM 2016]. ***(Continued)***

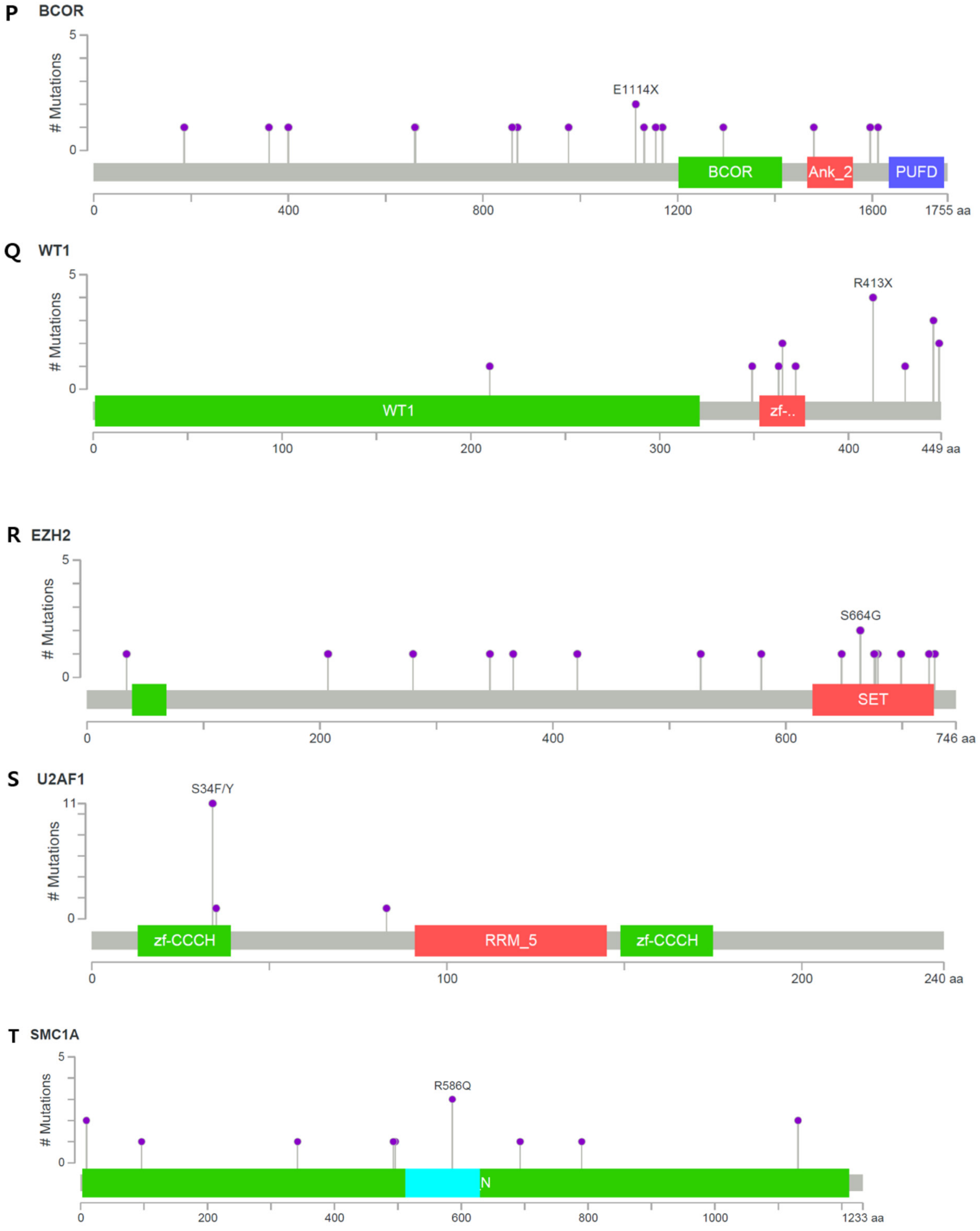

Supplementary Figure 2: Lollipop plot of the most frequently detected mutations **(A-V)** including TP53 **(W)**. We described the location of *TP53* mutation in **W(a)**; out cohort, **W(b)**; Metzeler's [Blood 2016] and, **W(c)**; Papaemanuil's data [NEJM 2016]. **(*Continued*)**

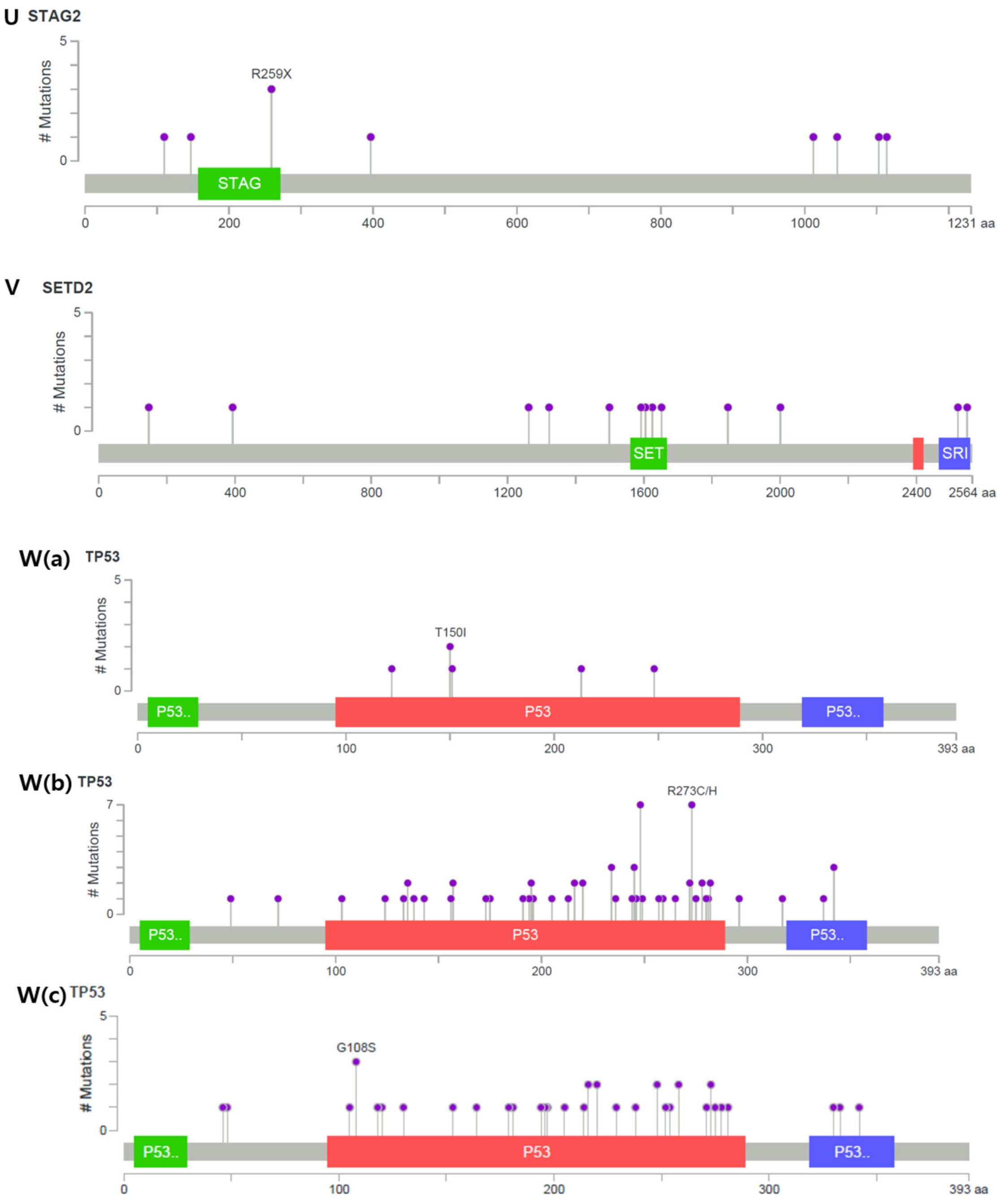

Supplementary Figure 2: Lollipop plot of the most frequently detected mutations **(A-V)** including *TP53* **(W).** We described the location of *TP53* mutation in **W(a)**; out cohort, **W(b)**; Metzeler's [Blood 2016] and, **W(c)**; Papaemanuil's data [NEJM 2016].

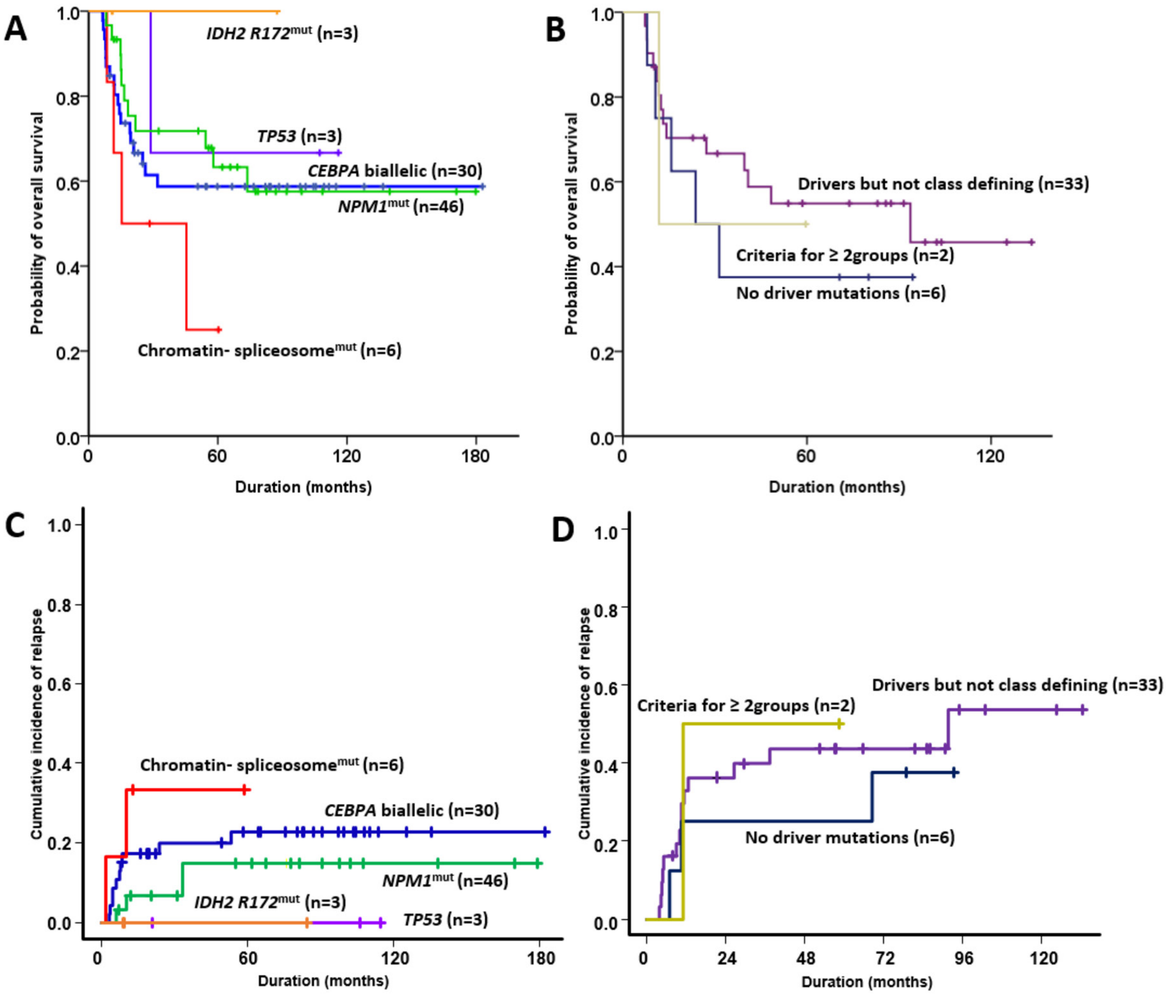

Supplementary Figure 3: Prognostic impact according to genomic classification in patients with normal-karyotype acute myeloid leukemia who underwent allogeneic stem cell transplantation. **(A, B)** overall survival, and **(C, D)** relapse incidence.

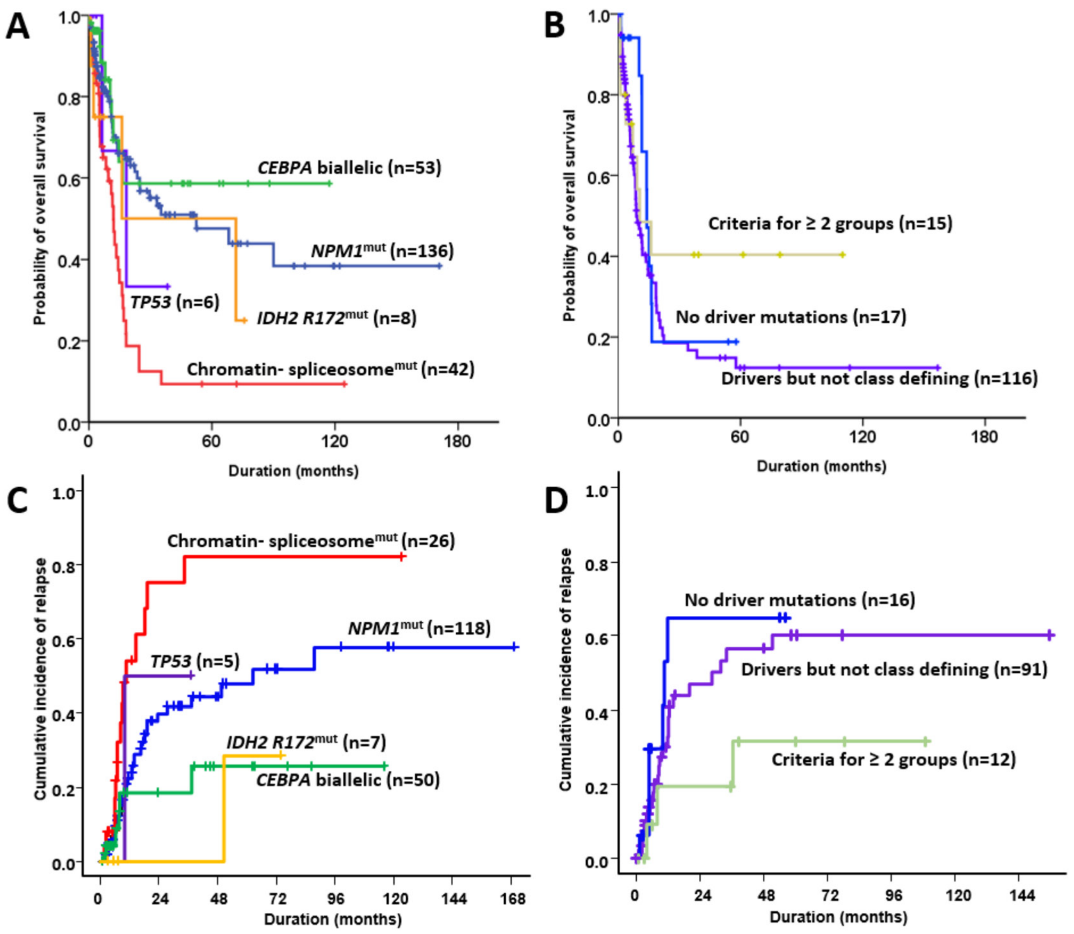

Supplementary Figure 4: Prognostic impact according to genomic classification in patients with normal-karyotype acute myeloid leukemia (patients undergoing allogeneic SCT were censored at the time of transplantation). (A, B) overall survival, and **(C, D)** relapse incidence.

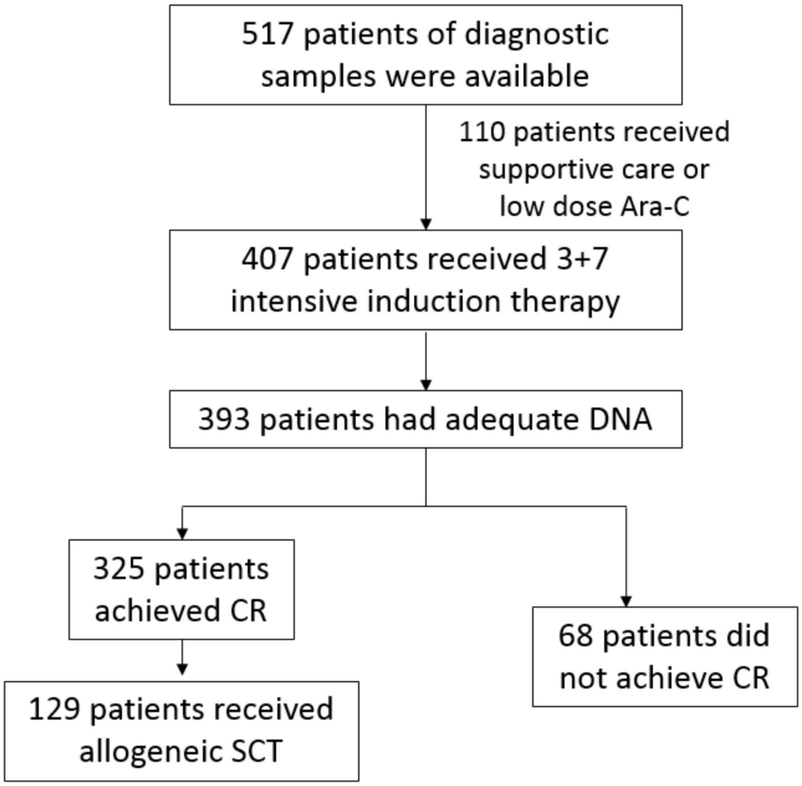

Supplementary Figure 5: Flowchart on patients' selection. Abbreviation: CR, complete remission; SCT, stem cell transplantation.

Supplementary Table 1: Gene panels of targeted deep sequencing and gene coverage.

See Supplementary File 1

Supplementary Table 2: Clinical characteristics of 129 patients with normal-karyotype acute myeloid leukaemia who received allogeneic stem cell transplantation

	No. of patients	%
**Gender, male**	64	49.6
**Age, Median (range, years)**	49 (15-64)	
**WBC, Median value (× 10^9/L), range**	25.7 (0.9-397.2)	
< 30.0×10^9/L	69	53.5
≥ 30.0×10^9/L	60	46.5
**Donor type**		
Matched related	78	60.5
Others	51	39.5
**Source**		
Bone marrow	26	20.2
PBSC	99	79.8
**Conditioning**		
Myeloablative	109	84.5
Reduced intensity	20	15.5
**GVHD prophylaxis**		
CsA ± MTX	85	65.9
FK506 ± MTX	44	34.1
**Use of T cell depletion**		
Yes	33 (23/10)[†]	25.6
No	96	74.4

[†] 23 patients received anti-thymocyte globulin and 10 patients received alemtuzumab for T cell depletion.

Abbreviations: WBC, white blood cells; PBSC, peripheral blood stem cell; GVHD, graft-versus-host disease; CsA, cyclosporine A; MTX, methotrexate.
